# Goal-oriented cognitive rehabilitation in early-stage dementia: study protocol for a multi-centre single-blind randomised controlled trial (GREAT)

**DOI:** 10.1186/1745-6215-14-152

**Published:** 2013-05-27

**Authors:** Linda Clare, Antony Bayer, Alistair Burns, Anne Corbett, Roy Jones, Martin Knapp, Michael Kopelman, Aleksandra Kudlicka, Iracema Leroi, Jan Oyebode, Jackie Pool, Bob Woods, Rhiannon Whitaker

**Affiliations:** 1 School of Psychology, Bangor University, Bangor, Gwynedd LL57 2AS, UK; 2 Cardiff University, Cardiff, UK; 3 University of Manchester, Manchester, UK; 4 Alzheimer’s Society, London, UK; 5 Kings College London, London, UK; 6 RICE (The Research Institute for the Care of Older People), Bath, UK; 7 London School of Economics, London, UK; 8 University of Bradford, Bradford, UK; 9 Jackie Pool Associates Ltd, Bishops Waltham, UK

**Keywords:** Alzheimer’s disease, Vascular dementia, Mixed dementia, Re-ablement, Quality of life, Cost-effectiveness

## Abstract

**Background:**

Preliminary evidence suggests that goal-oriented cognitive rehabilitation (CR) may be a clinically effective intervention for people with early-stage Alzheimer’s disease, vascular or mixed dementia and their carers. This study aims to establish whether CR is a clinically effective and cost-effective intervention for people with early-stage dementia and their carers.

**Methods/design:**

In this multi-centre, single-blind randomised controlled trial, 480 people with early-stage dementia, each with a carer, will be randomised to receive either treatment as usual or cognitive rehabilitation (10 therapy sessions over 3 months, followed by 4 maintenance sessions over 6 months). We will compare the effectiveness of cognitive rehabilitation with that of treatment as usual with regard to improving self-reported and carer-rated goal performance in areas identified as causing concern by people with early-stage dementia; improving quality of life, self-efficacy, mood and cognition of people with early-stage dementia; and reducing stress levels and ameliorating quality of life for carers of participants with early-stage dementia. The incremental cost-effectiveness of goal-oriented cognitive rehabilitation compared to treatment as usual will also be examined.

**Discussion:**

If the study confirms the benefits and cost-effectiveness of cognitive rehabilitation, it will be important to examine how the goal-oriented cognitive rehabilitation approach can most effectively be integrated into routine health-care provision. Our aim is to provide training and develop materials to support the implementation of this approach following trial completion.

**Trial registration:**

Current Controlled Trials ISRCTN21027481

## Background

There is a greater need than ever before to identify effective and beneficial interventions for people with early-stage dementia. There are thought to be over 750,000 people with dementia in the UK, a figure that is expected to have doubled by 2040 [1,2]. Current policy targets include ensuring early diagnosis and good quality early intervention for all and supporting people with dementia in living as full and active a life as possible [1]. Early diagnosis of dementia creates an opportunity to equip patients and carers to manage the disease effectively and to live well with dementia [3]. In this context early intervention offers the possibility of reducing or delaying the progression of functional disability, depression or behavioural difficulties, helping to maintain independence, supporting management of co-morbidity and hence avoiding or reducing hospitalisation, maintaining quality of life and ultimately delaying institutionalisation. At present, however, the chances of accessing early psychosocial intervention following a diagnosis of dementia are low, and research evidence regarding the efficacy of early psychosocial interventions remains limited. Research priorities set out by the Ministerial Advisory Group on Dementia Research (MAGDR) in 2011 indicate a need to evaluate the effect of psychosocial interventions for people with dementia living in the community, including those based on ‘re-ablement’, and to identify ways of improving quality of life for people with dementia and their carers.

Relatively little attention has been given to developing psychosocial early intervention approaches aimed at helping people to live well with dementia. Traditionally, efforts have focussed instead on attempting to address the underlying impairments in memory and other cognitive functions, which are a defining feature of early-stage dementia. A number of research studies have examined the potential of cognitive training to benefit people with dementia. Cognitive training involves repeated, structured practice on tasks targeting specific cognitive domains, such as working memory or attention. Evidence is mixed, with some studies reporting modest benefits and others reporting no benefits, but a Cochrane systematic review [4] found no evidence for significant benefits. Even where improvements on cognitive tasks assessing trained domains are reported, there is no evidence that these generalise to other areas, have any impact in the real-life context or offer any benefits as regards engagement in everyday activities [5]. That is to say, these approaches, which target underlying impairment, albeit with limited success, fail to reduce functional disability. Yet there is evidence for preservation of some degree of cognitive and neural plasticity in early-stage dementia [6], and it should be possible to harness this potential to deliver beneficial intervention effects. According to neuropsychological models of memory [7], while some cognitive functions (e.g. long-term episodic recall) are significantly impaired in early-stage Alzheimer’s disease, others are relatively spared (e.g. procedural memory for skills, routines and actions, semantic knowledge and implicit memory) [8], and people with early-stage dementia are capable of some new verbal and behavioural learning [9], although they are likely to require extra support to achieve it [10]. Consequently, there are possibilities for behaviour change to occur.

Conceptualising dementia within the framework of a disability model [11,12] highlights the distinction between the underlying impairment, resulting from pathological changes, and the resulting limitations on engaging in activity (disability) and restrictions on social participation (handicap). Activity limitation and participation restriction are not solely determined by the degree of impairment, but are subject to a range of personal, social and environmental influences. Negative influences can contribute to the development and maintenance of ‘excess’ disability [13], where the extent of functional disablement is greater than would be predicted by the degree of impairment; an example would be where an individual loses confidence, gives up previously enjoyed activities, and becomes socially withdrawn and depressed in reaction to receiving the diagnosis of dementia, with consequent effects on cognitive and functional ability. This is similar to Kitwood’s description of the way in which a negative, unsupportive social environment can undermine well-being for people with dementia [14]. Equally, positive influences can support optimal functioning and overcome some of the potential impact of impairment, enabling people to live well with dementia. A focus on addressing barriers to activity and participation, and encouraging adaptive behaviours, can therefore be expected to produce benefits for people with dementia and their family members.

Interventions that aim to reduce functional disability by targeting activity and participation, drawing on retained strengths to support adaptive behaviour, are typically described as forms of rehabilitation. Rehabilitation interventions aim to ‘enable people who are disabled by injury or disease to achieve their optimum physical, psychological [and] social well-being’ [15]. The rehabilitation of people who have cognitive, as opposed to purely physical, impairments is termed ‘cognitive rehabilitation’ (CR) [16]. Although rehabilitation is most often associated with non-progressive conditions such as brain injury, it is equally applicable to people with chronic and progressive conditions. There is considerable evidence for the efficacy of cognitive rehabilitation with a range of clinical groups [17]. Rehabilitation interventions are generally highly individualised, as clients have a diverse range of impairments, needs, circumstances and preferences. Central to the practice of rehabilitation is the identification of realistic and personally meaningful individual rehabilitation goals for each client and the development of tailored interventions to address these. Goal-based approaches have been applied in numerous conditions, including brain injury [18,19], stroke [20], neurological illness [21], physical disability [22] and chronic pain [23,24], as well as for frail older people [25]. Goals are, wherever possible, negotiated collaboratively between client and therapist. Such interventions may be regarded as inherently person-centred.

It has been suggested that rehabilitation provides a useful overarching conceptual framework for the care and support of people with dementia and for the design of interventions to meet their needs [26]. A few early examples of interventions that addressed meaningful individual goals relating to self-care or activity participation supported the possible utility of this approach [27,28]. Hence feasibility studies were undertaken to explore the application of cognitive rehabilitation to help people with early-stage dementia and their families manage the impact of the condition.

A series of studies using single-case experimental designs or small-group pre/post comparisons demonstrated that it was possible to identify meaningful personal goals and use evidence-based restorative or compensatory rehabilitation methods [29,30] to bring about behaviour change in these areas for people with early-stage dementia [31-34]. Restorative approaches build on retained abilities and use a range of instructional or prompting techniques to promote new learning or relearning, whether of information, habits or strategies; examples include the application of the spaced retrieval method to support retention of information [35]. Compensatory methods use a range of aids and adaptations to support functioning and overcome limitations resulting from cognitive impairments; examples include the use of memory books to support engagement in conversation [36]. Rehabilitation interventions for people with dementia need to offer practical benefits in daily life. In the context of cognitive impairment it is particularly challenging to ensure that learning and behavioural change generalises from one setting to another; to circumvent this obstacle, the interventions in our small-scale studies were carried out in the person’s everyday setting rather than in the clinic. The behavioural changes observed, although focused on specific targeted goals, led to wider benefits in everyday life; for example, learning names of other participants in a social club helped to maintain attendance and participation and reduced the risk of social isolation [31], and using a memory aid to reduce repetitive questioning reduced carer frustration and tensions between the participant and carer [32]. There was also some evidence for generalisation of the problem-solving approach to other everyday situations and challenges [32,33]. Gains were maintained for several months post-intervention, and one longer-term study demonstrated maintenance of gains up to 3 years post-intervention [37]. Further studies investigated the efficacy and applicability of specific memory rehabilitation techniques, such as errorless learning and spaced retrieval methods [38-40]. These findings were augmented by reports from other research groups [41,42]. A Cochrane systematic review found no randomised controlled trials of cognitive rehabilitation [4]. The findings from the feasibility studies, therefore, formed the basis for developing an intervention protocol that could be tested in a pilot randomised controlled trial [34].

The design of trials to evaluate the efficacy of rehabilitation interventions must take into account the fact that rehabilitation typically focuses on the attainment of highly individual goals that are functionally, socially and contextually relevant [43]. When evaluating service or programme outcomes in rehabilitation settings, goal attainment scaling has been used to identify goals and rate progress on a standardised scale [18,25,43,44]. However, where the focus is on treatment outcomes for the individual client, as opposed to overall efficacy of a multidisciplinary or multi-component programme, goal setting and goal achievement are more readily evaluated by means of client-centred approaches in which the client plays a central role in a collaborative goal-setting process, and the client’s perceptions of change serve as the primary outcome measure. The most widely used example of this approach is provided by the Canadian Occupational Performance Measure (COPM) [45], which offers a structured format for eliciting individual goals and a standardised means of rating goal performance and satisfaction with performance. There is evidence for the reliability, construct validity, sensitivity and responsiveness of this measure as well as for its clinical utility [20,46-51]. When using this measure in research, it is possible to elicit goals and performance ratings at baseline and to have participants in both treatment and control groups re-rate goal performance at follow-up. Where clients have cognitive impairments, it is helpful to supplement self-ratings with independent ratings made by professionals or caregivers for comparison purposes [20,50]. The goal-oriented approach accords with person-centred values in dementia care, allowing the person with dementia to engage in an intervention that is specifically tailored to his/her own needs and preferences, while also providing for a standardised group-level comparison. Therefore, more recently, the Bangor Goal-Setting Interview (BGSI) has been developed for research purposes [52]. This interview has similar aims to those of the COPM, but has been developed primarily as a research tool and incorporates a number of different features. The BGSI is based on the social cognitive theory of behaviour change [53] and on the concept of motivational interviewing [54], and the relevant domains of functioning in relation to which goals will be considered are selected according to the requirements of the given study.

The CR intervention is focussed on the identification and attainment of individual goals. The recently developed BGSI will be used in the GREAT trial. However, for the pilot trial, perceived goal performance, rated using the COPM, was selected as the primary outcome. A pilot trial of individual, goal-oriented CR, funded by the Alzheimer’s Society and published in the American Journal of Geriatric Psychiatry, was conducted in North Wales from 2005 – 2009 [55]. This was a single-site, single-blind randomised controlled trial comparing an eight-session CR intervention to relaxation therapy (RT), which involved equivalent therapist time and attention, and was expected to be pleasurable for participants without addressing the areas targeted in CR, and to treatment as usual (TAU). All participants received acetylcholinesterase-inhibiting (AChEI) medication and routine outpatient monitoring, and had access to the range of voluntary sector services available at the time in the area. The primary outcome was goal performance and satisfaction with performance. Sixty-nine participants were randomised by computer algorithm, independently operated by the North Wales Organisation for Randomised Trials in Health, Clinical Trials Unit (NWORTH CTU), to one of the three conditions (CR, *n* = 23; RT, *n* = 24; TAU, *n* = 22).

**Figure 1:**
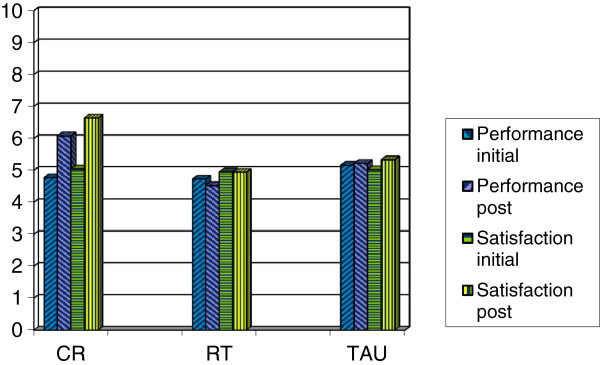
Effects of intervention on goal performance and satisfaction (COPM ratings) for participants in each condition in the pilot trial: significant improvements for CR and no change for RT or TAU.

Following intervention, goal performance and satisfaction ratings improved for the CR group and showed no change in the other two groups (see Figure 1). Analysis of covariance indicated a significant effect of CR on performance (*F*(2,58) = 7.880, *P* <0.001) and satisfaction (*F*(2,58) = 8.270, *P* < 0.001). For both measures, CR differed significantly to both RT and TAU (performance: 1.459 ± 0.936 for RT and 1.128 ± 0.989 for TAU; satisfaction: 1.686 ± 1.041 for RT and 1.193 ± 1.090 for TAU). For the CR group, achievement of therapy goals was corroborated in three ways through within-group analyses [56]. First, participants rated performance and satisfaction with performance for each goal targeted, recording significant increases (performance: *t*(25) = −3.742, *P* < 0.001; satisfaction: *t*(25) = −4.877, *P* < 0.001). Second, a separate therapist rating of goal performance was made at the start and end of therapy; this reflected significant improvements (*t*(25) = −8.027, *P* < 0.001). Third, a simplified goal attainment scaling procedure was used, whereby for each therapy goal behavioural indicators of full and partial attainment were established by the research team at the start of therapy and each goal was rated accordingly at the end of therapy. This classified 12 (46%) goals as fully implemented, 13 (50%) as partially implemented and 1 (4%) as not implemented. It was noted that many of the partially implemented goals would likely have been fully achieved given a little more time.

Secondary outcomes were evaluated in terms of effect sizes (Cohen’s d) for the CR group compared to the pooled control (RT and TAU) groups, as no differences were observed between the two control groups on any measure. Outcomes examined for the person with dementia were quality of life, mood and cognition (effect sizes are shown in Table 1). CR produced benefits in all three areas, and quality of life continued to improve at 6-month follow-up. It should be noted that for the most part mood was within the normal range at baseline and hence scope for improvement was limited. For carers, CR reduced stress and improved psychological well-being and quality of life (effect sizes are shown in Table 1), and in some cases these were maintained or continued to improve at follow-up.

**Table 1:** Effect sizes (Cohen’s d) on secondary outcome measures obtained in the pilot trial for the CR group compared to the pooled RT and TAU groups

**Measure**	**Post-intervention**	**6-month follow-up**
**Participants with dementia**		
Quality of life (QoL-AD)	0.24	0.29
Depression (HADS)	0.26	0.13
Anxiety (HADS)	0.21	0.11
Memory (RBMT)	0.37	0.08
Verbal fluency (FAS)	0.29	-
Sustained attention (TEA elevator counting)	0.76	-
Auditory selective attention (TEA ECD)	0.53	-
Visual selective attention (TEA map search 1 min)	0.11	-
Everyday problem-solving (ILS)	0.21	-
CARERS		
Stress (RSS)	0.54	0.27
Psychological well-being (GHQ)	0.51	0.11
Quality of life: social relationships (WHOQoL)	0.34	0.49
Quality of life: psychological (WHOQoL)	0.11	0.55
Quality of life: physical health (WHOQoL)	0.69	0.38
Quality of life: environment (WHOQoL)	0.46	0.08

*HADS* Hospital Anxiety and Depression Scale, *RBMT* Rivermead Behavioural Memory Test, *TEA* Test of Everyday Attention, *FAS* letter fluency for letters F, A, and S, *TEA ECD*, TEA elevator counting with distraction, *ILS* Independent Living Scales, *RSS* Relatives’ Stress Scale, *GHQ-12*, General Health Questionnaire.

The CR intervention was acceptable to, and well received by, participants and carers. Across all three groups, the attrition rate between randomisation and post-intervention assessment was 7%; five individuals discontinued because of physical illness (1), death (1), incorrect diagnosis (1) and self-withdrawal (2). Attrition between post-intervention assessment and 6-month follow-up was 12%; eight individuals were lost to follow up because of death (2), moving out of the area (3) and self-withdrawal (3). Thus, the overall rate of elective self-withdrawal for the trial was only 7% (2 each from CR and RT, and 1 from TAU).

This pilot trial demonstrated that participants with early-stage dementia can identify personally meaningful goals relating to managing everyday activities, and, with a modest amount of support from a therapist, make significant progress towards implementing these. Goal performance constituted a sensitive and specific measure of change. As performance and satisfaction ratings were closely associated, performance ratings should suffice in future work. The addition of carer ratings of performance would be informative. The trial provided valuable experience in collaborative identification of specific, measurable, achievable and realistic goals [56]. Results suggested that a slightly longer intervention might be advisable in order to fully establish and consolidate gains. The trial showed that CR can bring benefits with regard to cognition, well-being and quality of life for the person with dementia, as well as the well-being and quality of life of the carer. The lack of observed differences between the two control groups (RT and TAU) suggested that in a definitive trial TAU could be adopted as an appropriate comparison condition for CR. Findings from the pilot provided information about intervention parameters, outcomes and effect sizes that has informed the design of the planned multi-site trial presented below.

## Methods/design

**Figure 2:**
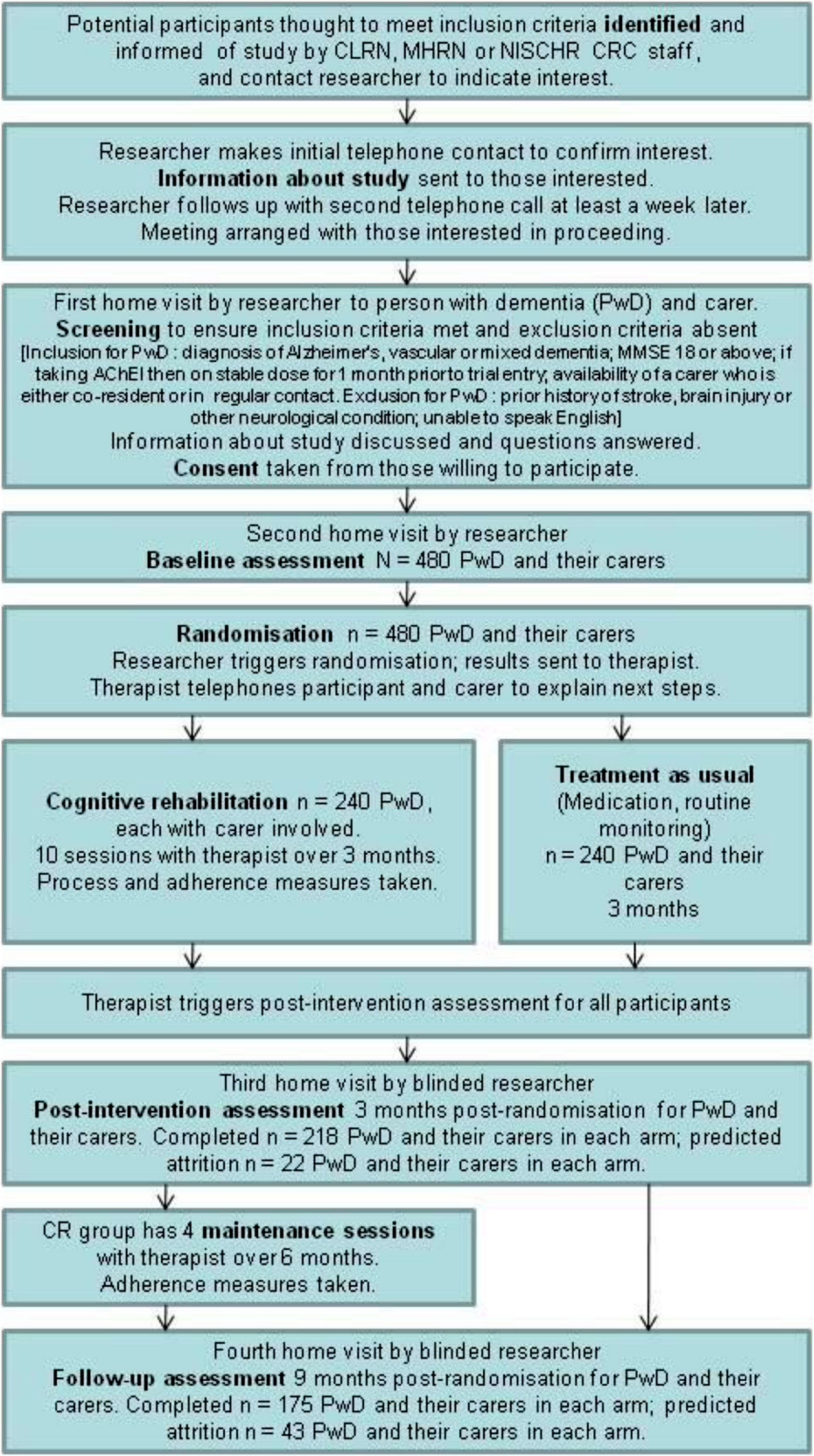
GREAT trial CONSORT-style flow chart.

This is a multi-centre single-blind randomised controlled trial comparing cognitive rehabilitation (CR) to treatment as usual (TAU) for people with early-stage Alzheimer’s, vascular or mixed dementia, with outcomes assessed at 3 and 9 months post randomisation. Participants will be recruited from memory clinics, old age mental health services and general practitioner (GP) practices. CR will be delivered in participants’ homes, with a carer involved where possible. The study will be run from the co-ordinating centre at Bangor University with six recruiting centres around the UK: North-West England, South-West England, West Midlands, London, South Wales and North Wales. At each recruiting centre, a part-time therapist (with an appropriate professional background, e.g. occupational therapy or psychology) will conduct the interventions, and a research assistant, blind to group allocation, will carry out assessments at baseline and at 3 and 9 months post-randomisation. Participants will be recruited to the trial between 1 April 2013 and 30 September 2015. A CONSORT-style flow chart for the trial is shown in Figure 2.

### The cognitive rehabilitation intervention

CR is an individualised approach for people with dementia (PwD) aimed at managing or reducing functional disability and maximising engagement and social participation. PwD and their carers work together with a health professional over a number of sessions to identify personally relevant goals and devise and implement strategies for achieving these. CR will be delivered in ten individual sessions over 3 months, followed by four maintenance sessions over 6 months. Carers will be involved in part of each session where possible. Involvement of a carer helps to ensure that skills are maintained and applied to novel situations and facilitates communication about how current or possible future difficulties might be managed.

Over the course of the 10 weekly sessions, participants with dementia will work in collaboration with the therapist to address personal rehabilitation goals. Drawing on the goals identified at baseline assessment, up to three behavioural goals will be operationalised, and strategies for addressing these will be devised and implemented. Goals will be introduced one at a time, in a flexible manner depending on rate of progress. Following introduction and modelling of strategies and skills during the therapy sessions, the participant and carer will work on the selected goal between sessions following an agreed schedule of activities. Progress with each goal will be reviewed and the strategies adopted will be adjusted as necessary on a weekly basis. Performance for each goal will be independently rated at the outset and in week 10 by the participant, carer and therapist, and the therapist will rate the extent of goal attainment following the sessions in week 10. Work on goals will be supplemented by the following components, designed to support the ability to make progress with the selected goals and address barriers to progress, which will be systematically introduced across the ten sessions:

1. Introduction of, and practice in applying, a solution-focused problem-solving approach by following a short sequence of steps to specify and test possible solutions.

2. Introduction of anxiety management strategies, building on participants’ existing strengths and preferences in this area, and practice in strategy use and application.

3. Monitoring of activity levels, leading to plans for increasing engagement in meaningful and enjoyable activity, and implementation of these plans.

4. Practice in strategies for maintaining or improving attention and concentration.

5. A review of compensatory strategy use (e.g. calendars, diaries, reminder systems) and development and implementation of plans for improving strategy use, which might include increasing the efficiency of existing strategies and introduction of new strategies.

6. A review of current use of restorative strategies for retaining new information or improving recall, and practice in key strategies (mnemonics, semantic association, spaced retrieval), enabling participants to identify a preferred strategy and apply this in everyday situations.

In addition, to ensure sensitivity to the wider context, where appropriate a discussion of carer well-being and strategies for managing stress will be initiated, and information will be provided about additional sources of support and help, with encouragement to access these. The four maintenance sessions will focus on supporting maintenance of gains and encouraging continued goal performance and strategy use. The therapist will re-rate the extent of goal attainment following the session in week 14.

The effects of the CR intervention will be compared to treatment as usual (TAU). In the pilot, CR was compared with TAU and with an attention placebo condition (relaxation therapy). There was no evidence of a difference between the two control groups, which suggests that TAU can serve as an appropriate comparator. For the CR group, the CR will be provided in addition to TAU, while the control group will receive only TAU and will have no contact with the research team between assessments. TAU will consist of acetylcholinesterase-inhibiting medication where prescribed, and any other services normally provided apart from specific programmes of cognitive rehabilitation or other cognition-focused intervention. TAU may include, for example, routine monitoring by the Memory Clinic, information provision, attendance at drop-in groups or support groups, or carer participation in support groups. Service receipt during the intervention period, including dementia-specific services, monitoring and interventions provided by Memory Clinics, will be documented for all participants. All participants will be free to access services such as those offered by the Alzheimer’s Society, and the extent of this will be recorded.

### Participant selection

Participants will be recruited from memory clinics, old age mental health services and GP practices, and will have been diagnosed with early-stage Alzheimer’s disease (AD), vascular dementia, or mixed AD and vascular dementia. For each participant, a carer (a family member or close friend who is either co-resident or in regular contact) will also be involved.

Inclusion criteria:

1. The participant must have been assigned an ICD-10 diagnosis of Alzheimer’s disease (AD), vascular dementia, or mixed AD and vascular dementia. AD accounts for 62% of dementia diagnoses, and vascular dementia for 17%, with mixed AD and vascular dementia accounting for a further 10% [2]. These categories together capture 89% of those diagnosed with dementia. There is no reason to assume that people with rarer subtypes of dementia, including dementia with Lewy bodies (4%), fronto-temporal dementia (2%) and Parkinson’s dementia (2%), could not benefit from CR, but these forms of dementia have specific features that would require a distinct approach. For this reason, and because numbers are likely to be too small to allow for subgroup analysis, we are not proposing to include these groups in the current trial.

2. The participant must be in the early stages of dementia, as indicated by an MMSE score of 18 or above. This is to ensure that participants recruited to the trial have a level of cognitive functioning that is sufficient to allow them to complete the selected outcome measures without undue difficulty for the duration of their participation in the study. Use of a cutoff point, while inevitably somewhat arbitrary, provides protection for people who may be overly burdened by the assessments. The selected cutoff of a score of 18 on the MMSE is frequently used in research studies and worked well in the pilot trial. We have not placed any upper limit on the MMSE score, since it can be expected that a small proportion of people meeting diagnostic criteria for dementia will have high MMSE scores [57].

3. If taking acetylcholinesterase inhibitors, the participant must have been receiving a stable dose for 1 month prior to trial entry, and there should be no intention to change the dose over the period of participation in the study unless clinically indicated. This is to ensure that intervention effects are not confounded by changes in medication status.

4. The participant must have a carer who is willing to participate. While having a carer is not an essential prerequisite for receiving a CR intervention, it is important for the purposes of research to obtain an informant perspective on the effects of the intervention, and in this trial carers will be asked to provide an independent rating of goal performance. It is also important to determine the effects of the intervention on carer well-being; positive effects on the carer are likely to bring added benefits in the longer term for the person with dementia.

5. The participant must be able to give informed consent. People in the early stages of dementia are normally expected to have capacity to consent to participation. When recruiting participants, the research team will use a checklist to ensure that all relevant information is considered and that the participant is able to give informed consent. While CR principles may be applied at any stage of dementia, the intervention to be tested here involves engaging the person with dementia in a collaborative process of identifying and addressing personally meaningful goals, and therefore the participant needs to be able to understand this and to make a positive choice to take part.

Exclusion criteria:

1. Participants will be excluded if they have a prior history of stroke, brain injury or other significant neurological condition. Such conditions would be expected to affect cognitive, behavioural and emotional functioning, and people who have one of these conditions prior to developing dementia would have additional rehabilitation needs. While such individuals might benefit from CR, their inclusion would represent a potential confounding factor.

2. Participants will be excluded if they are unable to speak English. This criterion is applied for practical reasons, because the standardised outcome measures we plan to use are only available in English. No official data are available to indicate what proportion of the UK population cannot speak English; while it is estimated that about 3% of the population use a language other than English at home, with over 100 different languages represented (source: The National Centre for Languages), many of the individuals concerned also speak English. The time and costs involved in translating standardised measures and providing interpreters for assessment and therapy sessions would be substantial. However, we predict that very few individuals would be excluded from participation because of inability to communicate in English.

### Ethical considerations

The study has been reviewed by the North Wales Research Ethics Committee–West, which issued a favourable opinion on 25 June 2012 (Reference 12/WA/0185) and has been approved by the Bangor University School of Psychology Research Ethics Committee.

Based on previous findings, participants randomised to receive CR may be expected to derive some benefits in terms of managing everyday activities and general well-being. Their caregivers may also be expected to show reduced stress and improved well-being. Availability of evidence from a definitive trial may be expected to have a positive influence on the future provision of interventions to support people with early-stage dementia and their carers. Previous findings also suggest that participants randomised to TAU are expected to show little or no change; thus, they will not be harmed by this allocation. As the trial will provide the first evidence from a large-scale trial regarding the benefits of CR, it cannot at this stage be considered unethical to withhold this treatment from the control group, and the control group will still have access to the care typically provided by memory clinics and GPs, and to voluntary sector services.

There are no known risks or side effects associated with CR. It is possible that some participants may find it challenging to confront their difficulties, but the therapist will provide support as they engage in this process, and the intervention protocol incorporates attention to managing emotional reactions. Neither the feasibility studies nor the pilot trial have suggested that this represents a significant risk to participants. The research team will be trained to be alert to any concerns about participants’ well-being. If there are serious concerns about a participant, these will be referred, wherever possible with the permission of the individual concerned and the carer, to the clinician responsible for the participant’s care.

Participants with early-stage dementia, and carers, will be fully informed prior to entry into the trial about the intervention and about the current state of knowledge regarding possible benefits and risks, and this information will be updated if additional evidence becomes available during the course of their participation.

Informed consent will be obtained from all participants and carers. People with early-stage dementia are expected to have the capacity to consent to participation. While consent provided at the outset provides an initial mandate for entry to the trial and commencement of trial procedures, consent is an ongoing process, and this is crucial for psychosocial interventions where participants’ active engagement is required. Therefore, research assistants and therapists will be trained to monitor ongoing consent and to respond appropriately to any indication of a possible withdrawal of consent. As participants will be in the early stages of dementia, loss of capacity to consent during the course of participation is expected to be infrequent. However, on entry to the trial participants will be asked whether, should they lose capacity to consent, they are willing to continue to be included in the trial and to have their data used.

Personally identifiable information will be retained only until publication of the trial report unless the participant has consented to retention of details for potential further follow-up, while anonymised data will be retained for 5 years after publication unless a longer period is required by the Research Ethics Committee or other regulatory authorities. Consent forms will be retained for 25 years following trial closure.

The governance and management of the study will be undertaken within the Department of Health Research Governance Framework for Health and Social Care (2nd edition, 2005). This will ensure the highest standards of clinical research, covering scientific quality, ethical standards and all related management issues. The trial will adhere to the Standard Operating Procedures (SOPs) of NWORTH CTU [58] for all trial and data management, statistical and regulatory matters. This is not a clinical trial of an investigational medical product (CTIMP) and therefore it does not come under the provisions of the Medicines for Human Use (Clinical Trials) Regulations (2004).

All research staff and therapists will undergo training in Good Clinical Practice with regard to the conduct of clinical trials. Trial-specific training requirements will be addressed throughout the study period and regularly reviewed. Orientation and project-specific training will be provided for CLRN, MHRN and NISCHR CRC staff.

### Sample size

Power calculations and attrition rates are based on findings from the pilot trial. We wish to confirm the finding that the primary effectiveness outcome of goal performance was improved in the treatment group [45]. The difference observed in the pilot was large (standardised effect >1 at post-intervention assessment). However, we also wish to detect any effect sizes in the order of 0.3 for important secondary outcomes, as we judge that this will give confirmation of effects that are large enough to have substantive clinical benefits. For the present study, intervention length has been increased and now includes a maintenance phase in order to further strengthen demonstrable effect sizes in secondary outcomes. We have elected to be conservative in all aspects of our estimate of power, and we have made a larger estimation of potential attrition than the <20% observed in that study, based on the 27% rate observed in the recent REMCARE trial [59]. To achieve 80% power to detect a medium effect size of 0.3, with alpha 0.05, in primary and secondary outcomes, 175 PwD, with their carers, need to complete the trial in each arm. Adjusting for potential attrition, we aim to randomise 480 PwD, each with a carer. There will be one practitioner involved at each centre, and we will test for differences between sites in the analysis. If there is a change of practitioner at one or more centres during the course of the trial we will also test for practitioner effects.

### Outcome measures

#### (1) Primary outcome measure

##### Bangor Goal-Setting Interview (BGSI)

The BGSI [52] provides a structured format for the goal-setting process. The interview proceeds in three stages. First, relevant domains of functioning are discussed in turn with a view to eliciting issues that might form the basis for behavioural goal-setting. For each area, the participant rates perceived importance of making changes in this area, and readiness to make changes in this area, on a 1 – 10 scale (where 1 is not at all important/not at all ready and 10 is extremely important/completely ready). Once all areas have been discussed, the second stage involves revisiting each area in turn and negotiating specific behavioural goals that conform to ‘SMART’ principles (specific, measurable, achievable, realistic and time-delineated). Additionally, goal attainment indicators are specified, providing clear descriptors of what would constitute 25%, 50% and 75% goal attainment. In the final stage of the interview, the participant is asked to rate, for each of the goals that have been identified, current performance and satisfaction with performance on a 1 – 10 scale (where 1 is unable to perform/extremely dissatisfied and 10 is able to perform perfectly/extremely satisfied). Mean scores for performance and satisfaction with performance across goals are calculated by dividing in each case the sum of the scores for all goals identified by the number of goals set. For the present study, details of the goals identified in the baseline BGSI assessment will be provided to the therapist for each participant allocated to the CR condition and will form the starting point for the therapist’s intervention. At follow-up assessment, the participant re-rates current performance and satisfaction with performance for each goal so that changes in ratings can be examined. The interviewer elicits information about current performance and uses the previously specified goal attainment indicators to rate the extent of progress towards achieving the goal. There is scope within the interview format for informant ratings to be obtained; in this study, carer ratings of performance and descriptions of goal attainment will be elicited at baseline and follow-up for comparison purposes.

#### (2) Secondary outcome measures for participants with dementia

##### DEMQOL

DEMQOL [60] has been designed to assess health-related quality of life of people with dementia across the full range of severity and subtypes, and shows high internal consistency (Cronbach’s alpha 0.87) and good test-retest reliability (ICC 0.76) in people with mild to moderate dementia. It consists of a 28-item interviewer-administered questionnaire for the person with dementia and a 31-item interviewer-administered questionnaire on which the caregiver provides proxy ratings. These may be used together or separately. In this study, only self-ratings by the person with dementia will be taken. An algorithm has been developed to generate quality-adjusted life year (QALY) scores from DEMQOL scores for use in economic evaluation [61].

##### Generalised Self-Efficacy Scale (GSES)

The ten-item Generalised Self-Efficacy Scale (GSES) [62] was created to assess a general sense of perceived self-efficacy, the potential to influence one’s situation through one’s own actions. Responses are made on a 4-point scale. Responses to all ten items are summed to yield the final composite score with a range from 10 to 40. Cronbach’s alphas range from 0.76 to 0.90 [63].

##### Hospital Anxiety and Depression Scale (HADS)

The HADS [64] contains 14 items forming two subscales: anxiety and depression. Each item is rated on a 4-point scale, giving maximum scores of 21 for anxiety and for depression. Scores of 11 or more on either subscale are considered to be a significant ‘case’ of psychological morbidity, with scores of 8–10 classified as ‘borderline’ and 0–7 ‘normal’. The HADS has been employed and validated in studies of people with dementia and carers [65,66].

##### Brief cognitive assessment battery

This will consist of brief tests of memory, attention and executive function, suitable for people with early-stage dementia, each taking less than 5 min to administer: (1) Memory: Rivermead Behavioural Memory Test (RBMT) [67], story recall subtest. The RBMT is a well-established, ecologically valid test of everyday memory. In the story recall task, the researcher reads out a short story, similar to a brief report of a newsworthy event in a daily newspaper, and the participant is asked for immediate and delayed (after 20 min) recall of the content. Recall is scored following a standard protocol (inter-rater reliability > 0.9) with a maximum possible score of 21 for the immediate and for the delayed component. Four equivalent versions are available to permit reassessment without the risk of practice effects; practice effects are not anticipated with test-retest intervals of 3 and 6 months, but as a precaution a different version will be used at each time point. Raw scores will be used in the analysis as they provide a greater range than the condensed standardised profile score that is used in calculation of the overall RBMT score. (2) Attention: Test of Everyday Attention (TEA) [68], elevator counting and elevator counting with distraction subtests. The TEA is a well-established, ecologically valid test of everyday attention, with subtests assessing different components of attention. The elevator counting subtest assesses sustained attention. Participants are required to count a short string of monotonous tones and give the total number. Seven strings are presented, and the total score is the number of strings correctly counted. The elevator counting with distraction subtest assesses auditory selective attention. Further strings of tones are presented, this time also including distractor (high-pitched) tones that are not to be counted. The total score is the number of strings correctly counted. Three equivalent versions of each subtest are available to permit reassessment without the risk of practice effects; as above, practice effects are not anticipated but as a precaution a different version will be used at each time-point. (3) Executive function: Letter fluency subtest of the Delis-Kaplan Executive Function System (D-KEFS) [69]. D-KEFS consists of a set of standardised tests of executive function. The verbal letter fluency task evaluates the executive subdomains of initiation, response generation and inhibition [70] and draws on semantic memory and language ability. In this task, the participant is asked to list as many words as possible beginning with a specific letter of the alphabet in a 1-min period, excluding proper nouns and repetitions. Three letters, F, A and S, are used. The total number of correct responses to the three letters is used in analysis. This task has been extensively examined in people with early-stage dementia [71]. Evidence suggests that even in healthy participants there are no practice effects for most components of this task even at test-retest intervals of less than 2 weeks; there are minimal practice effects for the switching component with test-retest intervals of less than 2 weeks, but not with longer intervals.

#### (3) Secondary outcome measures for the carer

##### Relatives’ Stress Scale (RSS)

The RSS [72] is a 15-item dementia-specific measure of caregiver stress with items rated on a 5-point Likert scale and summed. A higher overall score indicates higher levels of caregiving-specific stress.

##### EuroQOL (EQ5D)

The EQ-5D [73] is a standardised measure of health status and health outcome, applicable to a wide range of health conditions. In the first section, the respondent is asked to select one of three options for each of five dimensions: mobility, self-care, usual activities, pain/discomfort and anxiety/depression. For each dimension, the three response options are coded on a 3-point scale from 1 (no problem) to 3 (unable to perform/extreme problem). This yields a descriptive profile (e.g. 11232) across the five dimensions. The second part of the measure is a visual analogue scale for self-rating of health-related quality of life (‘your health state today’). This measure is included because the EQ5D score will be used to generate QALY scores using societal weights.

##### WHO Quality of Life – BREF(WHOQOL-BREF)

The WHOQOL-BREF [74] is a 26-item scale assessing perceived quality of life, giving scores in four domains: environment, social relationships, psychological and physical health.

#### (4) Service utilisation

##### Client Services Receipt Inventory (CSRI)

The CSRI [75] provides a template that can be adapted to the needs of each specific study. Respondents are asked about their use of health care services for a period preceding baseline assessment and during the study period. The questions cover contact with a range of health and social care professionals, prescription of medications, hospital appointments and stays, participation in local authority funded activities such as day centres, participation in activities run by voluntary organisations and the contribution of informal carers. Questions to examine the nature and extent of any dementia-specific treatment received from the Memory Clinic will be included.

#### (5) Demographic and background information for the person with dementia and carer

Details such as gender, age, relationship between person with dementia and carer and whether they live together, age of onset of dementia, educational level, social class and co-morbidities will be collected. This will allow us to examine effects of demographic and social variables on treatment efficacy.

#### (6) Process measures for the CR group

For the cognitive rehabilitation group, process measures will be taken to provide convergent evidence about change in goal performance. In-session parallel ratings of goal performance by participant, carer and therapist will be made when each goal is introduced and in session 10. A simplified goal attainment scaling procedure [43] will be applied, as described for the pilot trial; clearly specified behavioural indicators of full and partial goal achievement will be defined when each goal is introduced, and progress according to these criteria will be rated by the therapist following session 10 and again following session 14.

#### (7) Therapist adherence to protocol

Therapist adherence to the treatment protocol will be monitored through therapy logs and structured supervision sessions. Therapists will receive monthly telephone supervision and face-to-face supervision meetings will be held every 3 months. Therapy logs reporting session content (with participant details anonymised) will be submitted to the supervisor for scrutiny prior to supervision sessions.

#### (8) Treatment compliance

Treatment compliance will be indexed by the number of sessions completed for each participant.

### Procedure

Initial identification of participants will be made by National Institute for Health Research (NIHR) Comprehensive Local Research Network (CLRN) and Mental Health Research Network (MHRN) staff in England and National Institute of Social Care and Health Research Clinical Research Collaboration (NISCHR CRC) staff in Wales. Participants will be contacted by or on behalf of the clinician responsible for their care and invited to respond directly to the research team to express an interest in finding out more about the study. Interested participants and carers will then be contacted by telephone by the local research assistant, who will provide additional information and send out written details. This will be followed by a further telephone call; for those interested in finding out more, a meeting will be arranged at which the research assistant will explain the study, answer any questions they may have, re-check eligibility and ensure that the person with dementia has the capacity to consent. Consent from the participant and the carer will be taken at, or following, this visit.

At each centre, once participants have given informed consent, they will be visited by the research assistant who will conduct the baseline assessment. Following this assessment, the research assistant will trigger randomisation. Results of the randomisation will be sent to the therapist, who will telephone the participant and the carer to explain the next steps. Participants allocated to CR will receive 10 weekly visits from the therapist over a 3-month period. The therapist will trigger the post-intervention assessment for all participants. The research assistant will visit each participant to conduct the assessment. Following the post-intervention assessment, participants in the CR group will receive four maintenance sessions with the therapist over a 6-month period. The research assistant will visit all participants 6 months after the post-intervention assessment to carry out the final 6-month follow-up assessment. All primary and secondary outcome measures, and service utilisation measures, will be administered at each assessment point.

After consent and baseline assessment, participants will be individually randomised. Randomisation to GREAT will be achieved by secure web access to the remote randomisation centre, NWORTH CTU, at Bangor University. This system will be set up, maintained and monitored independently of the trial statistician or other trial staff. The randomisation will be performed by dynamic allocation [76] to protect against subversion while ensuring that the trial maintains good balance to the allocation ratio of 1:1 both within each stratification variable and across the trial. Participants will be stratified by centre, gender, age (under 75 *vs.* 75 and above) and MMSE score (under 24 *vs.* 24 and above). For validation purposes, additional information will be taken including the participant’s trial number, initials, and date of birth, and details of the person requesting the randomisation.

This is a single-blind trial. The researchers taking the measures will be blind to allocation, as will the data analysts. The importance of maintaining blinding will be emphasised in the training for both research assistants and therapists. As the participants are not blind to their treatment, at post-intervention and follow-up assessments participants will be specifically asked not to comment on the nature of their involvement in the study and not to reveal to the researcher whether or not they were visited by the therapist. Following each assessment, the blinded researcher will note to which condition s/he thought the participant had been allocated and how certain s/he was of the allocation. Sensitivity analyses will be performed to determine whether this knowledge affected participant scores. If there is evidence to suggest that consequential bias is present, the analysis will be adjusted to counteract this effect.

Other protection from bias will include the method of allocation to groups. The randomisation will be performed independently of the data analysis team by the CTU using a dynamic, stratified, web-based system designed to protect from bias by the unpredictability of the algorithm and the security of the web-based programme. Blinding will be maintained by automatic generation of randomisation codes and distribution via e-mail directly to the therapists responsible for implementing the treatment. Further bias protection will come from a “treatment as allocated” analysis, which will be the principal analysis performed on both primary and secondary outcomes. Treatment compliance measures will be restricted to inclusion in secondary analyses.

We will collect basic anonymised demographic data and reasons for not progressing to trial participation for all those people identified as warranting screening and invitation to the trial but declining to be screened or to participate. These data will be reported on a CONSORT diagram, together with information on the amount and nature of missing data, to enable readers to assess bias arising from recruitment or acceptability issues within the trial.

### Statistical analysis

Demographic and baseline data will be fully described and all outcome data will be analysed and reported. Significance will be assumed to be 5% throughout, and 95% confidence intervals will be quoted. All data will be anonymised and coded so that data collection and statistical analysis are blind to treatment allocation. The code will be broken only after the primary analysis has been completed. A fully pre-specified analysis plan will be prepared prior to the data being released to analysts. The analysis will be performed on a “Treatment as Allocated” principle to ensure protection against unintended bias. The data will be fully imputed in line with the pre-defined statistical analysis plan to minimise data loss due to missing values or time points. Sensitivity analyses (best case/worst case) will be performed to assess the influence of differing imputation assumptions. All trial reporting will be CONSORT-compliant [77].

For each outcome measure, at both post-intervention and follow-up, three analyses will be presented, the first two being unadjusted and adjusted treatment-as-allocated analyses and the third a treatment received analysis:

1. An unadjusted two-sample *t*-test by allocation group.

2. An analysis of covariance with baseline score and stratification variables as the covariates and allocated group as the condition factor. Between-group effect sizes with confidence intervals will be calculated using Cohen’s d. Centre will be added as a fixed factor to test and quantify any site-specific effects, and if the number of practitioners is greater than the number of centres, practitioner will be added as a random factor.

3. A repeat of analysis 2 with treatment compliance factored in.

If CR is shown to be effective, additional forward stepping regression modelling will be undertaken to identify factors important in maximising the observed effects. Factors that will be investigated will include diagnostic category, medication status, educational level, social class, caregiver relationship to the person with dementia (spouse, adult child, other), and whether or not the carer is residing with the person with dementia.

For the cost-effectiveness analysis (CEA), service utilisation and carer input data will be collected using the CSRI. Unit costs will be attached to service use measures (from national reference costs, the PSSRU compendium [78] or calculated anew), CR costed in liaison with providers, and carer inputs valued using opportunity and replacement cost options. The CEA will look at changes over 9 months from each of two perspectives (health and social care; societal) in four analyses: cost of achieving an incremental change in BGSI; cost of achieving incremental changes in self-efficacy for participants with dementia; cost of achieving incremental QALY gains for participants with dementia; cost of achieving incremental QALY gains for carers. Incremental cost-effectiveness ratios will be computed as required and acceptability curves plotted for a range of willingness-to-pay values. Net-benefit regressions will make it possible to control for site, baseline outcome measures (where appropriate) and baseline costs, as well as gender, age and MMSE score. Sensitivity analyses will be conducted to test for different assumptions in the attachment of costs. We will also estimate the investment costs and net costs to the NHS and the social care system of making CR available nationally.

### Research governance

The research will be sponsored by Bangor University. The sponsor will ensure that appropriate indemnities are in place. The research will be overseen by a Trial Steering Committee and a Data Monitoring and Ethics Committee. Safety data will be routinely reported to the trial Data Monitoring and Ethics Committee (DMEC), and any suspected unexplained adverse reactions (SUSARs) noted will be reported to both the DMEC and the trial sponsor within established timeframes. Given the nature of the intervention, no interim analyses are planned, but such analyses may be requested at any time by the DMEC.

The issue of potentially competing studies will be monitored carefully. At the time of developing the protocol, only one study that could be perceived as potentially competing had been identified by local networks; that study was recruiting people with lower MMSE scores, and recruitment was due to be completed by May 2013. GREAT is a pragmatic trial and therefore participation would not preclude involvement in other clinical trials *per se*, unless those trials involved cognition-focused intervention. However, participant burden and inclusion/exclusion criteria in other, fastidious trials may preclude such dual participation. Each local PI will review any situation where there may be a conflict of recruitment pathways between trials to ensure that all potential participants are offered the most suitable option based on closest fit to eligibility criteria and participant preference.

### Service user involvement

Service users have been involved in the feasibility and pilot stages of the research leading to the development of this trial. The pilot trial benefitted greatly from the involvement of Alzheimer’s Society Research Network Volunteers. During the development of the present study we again sought, and took into account, the views of Alzheimer’s Society Research Network Volunteers and of service users contributing to the Dementia and Neurodegenerative Diseases Research Network (DeNDRoN) Patient and Public Involvement (PPI) programme. PPI for the GREAT trial will be provided through a partnership with the Alzheimer’s Society; this will ensure that service users are fully involved with the design, delivery and dissemination of the research. Service users will be consulted at each stage of the trial to ensure optimal tailoring of study protocols and procedures. To ensure that PPI is integrated throughout the study, two service user representatives will sit on the Trial Steering Committee. The Alzheimer’s Society Research Network will also contribute to dissemination activities towards the end of the study, ensuring that outcomes are communicated to lay audiences and policy-makers.

### Implementation within routine health-care provision

This will be the first multi-centre trial of individualised, goal-oriented cognitive rehabilitation for people with early-stage dementia. The CR approach offers a practical means of engaging people with dementia and carers in an early intervention process that aims to reduce functional disability and maximise engagement and participation, contributing to the possibility of living well with dementia. This approach can readily be offered by memory clinics in the period following diagnosis. Several UK memory services have already expressed interest in implementing CR. CR is also becoming acknowledged internationally; for example, it has recently been authorised for insurance reimbursement in Belgium, and has been conducted with the aid of trained volunteers in Canada. People with dementia have themselves begun to advocate for a rehabilitation approach [79]. The trial will provide the necessary evidence base to extend these developments, if the findings demonstrate that CR is indeed both clinically beneficial and cost-effective. Towards the end of the trial, we will build on the experience gained at each site to demonstrate how the CR approach can most effectively be integrated into routine health-care provision. Once recruitment for the trial comes to an end, we will develop materials and offer training for clinical teams and therapists, as well as preparing information for people with dementia, carers and the general public. Information about good practice in this area will be disseminated through a range of routes.

## Trial status

The GREAT trial started on 1 October 2012 and will be recruiting participants from 1 April 2013 to 30 September 2015. The end date for the trial is 31 December 2016.

## Abbreviations

GREAT: Goal-oriented cognitive rehabilitation in early-stage dementia: study protocol for a multi-centre single-blind randomised controlled trial; PwD: People with dementia; AD: Alzheimer’s disease; CEA: Cost-effectiveness analysis; COPM: The Canadian Occupational Performance Measure; NWORTH CTU: North Wales Organisation for Randomised Trials in Health, Clinical Trials Unit; SOPs: Standard Operating Procedures; NIHR: National Institute for Health Research; CLRN: Comprehensive Local Research Network; MHRN: Mental Health Research Network; NISCHR CRC: National Institute of Social Care and Health Research Clinical Research Collaboration; DeNDRoN: Dementia and Neurodegenerative Diseases Research Network; PPI: Patient and Public Involvement; DMEC: Data Monitoring and Ethics Committee; BGSI: Bangor Goal-Setting Interview; GSES: Generalized Self-Efficacy Scale; HADS: Hospital Anxiety and Depression Scale; RBMT: Rivermead Behavioural Memory Test; TEA: Test of Everyday Attention; D-KEFS: Delis-Kaplan Executive Function System; RSS: Relatives’ Stress Scale; EQ5D: EuroQOL; WHOQOL-BREF: WHO Quality Of Life - BREF; CSRI: Client Services Receipt Inventory.

## Competing interests

The authors declare that they have no competing interests.

## Authors’ contributions

LC: study concept; preparation of study protocol; drafting of manuscript. ABa: preparation of study protocol; critical review of manuscript. ABu: preparation of study protocol; critical review of manuscript. AC: preparation of study protocol; critical review of manuscript. RJ: preparation of study protocol; critical review of manuscript. MKn: preparation of study protocol; critical review of manuscript. MKo: preparation of study protocol; critical review of manuscript. AK: critical review of manuscript. IL: critical review of manuscript. JO: preparation of study protocol; critical review of manuscript. JP: preparation of study protocol; critical review of manuscript. BW: preparation of study protocol; critical review of manuscript. RW: preparation of study protocol; critical review of manuscript. All authors read and approved the final manuscript.
